# MHY440, a Novel Topoisomerase Ι Inhibitor, Induces Cell Cycle Arrest and Apoptosis via a ROS-Dependent DNA Damage Signaling Pathway in AGS Human Gastric Cancer Cells

**DOI:** 10.3390/molecules24010096

**Published:** 2018-12-28

**Authors:** Jung Yoon Jang, Yong Jung Kang, Bokyung Sung, Min Jeong Kim, Chaeun Park, Dongwan Kang, Hyung Ryong Moon, Hae Young Chung, Nam Deuk Kim

**Affiliations:** College of Pharmacy, Molecular Inflammation Research Center for Aging Intervention (MRCA), Pusan National University, Busan 46241, Korea; jungyoon486@hanmail.net (J.Y.J.); biogreen80@hotmail.com (Y.J.K.); auvers1516@gmail.com (B.S.); nanum105@hanmail.net (M.J.K.); sauryyyy@pusan.ac.kr (C.P.); 3607@pusan.ac.kr (D.K.); mhr108@pusan.ac.kr (H.R.M.); hyjung@pusan.ac.kr (H.Y.C.)

**Keywords:** MHY440, topoisomerase inhibitor, cell cycle arrest, apoptosis, gastric cancer cells

## Abstract

We investigated the antitumor activity and action mechanism of MHY440 in AGS human gastric cancer cells. MHY440 inhibited topoisomerase (Topo) Ι activity and was associated with a DNA damage response signaling pathway. It exhibited a stronger anti-proliferative effect on AGS cells relative to Hs27 human foreskin fibroblast cells, and this effect was both time- and concentration-dependent. MHY440 also increased cell arrest in the G2/M phase by decreasing cyclin B1, Cdc2, and Cdc25c, and upregulating p53 and p73. MHY440 induced AGS cell apoptosis through the upregulation of Fas-L, Fas, and Bax as well as the proteolysis of BH3 interacting-domain death agonist and poly(ADP-ribose) polymerase. It also contributed to the loss of mitochondrial membrane potential. The apoptotic cell death induced by MHY440 was inhibited by pretreatment with Z-VAD-FMK, a pan-caspase inhibitor, indicating that apoptosis was caspase-dependent. Moreover, the apoptotic effect of MHY440 was reactive oxygen species (ROS)-dependent, as evidenced by the inhibition of MHY440-induced PARP cleavage and ROS generation via *N*-acetylcysteine-induced ROS scavenging. Taken together, MHY440 showed anticancer effects by inhibiting Topo I, regulating the cell cycle, inducing apoptosis through caspase activation, and generating ROS, suggesting that MHY440 has considerable potential as a therapeutic agent for human gastric cancer.

## 1. Introduction

Gastric cancer (GC) is the third leading cause of cancer death in both sexes worldwide, and it is especially common in less developed countries [1]. In Asia, GC is the third-most common cancer after breast cancer and lung cancer, and it is the second most frequent cause of cancer death after lung cancer. Although the incidence and mortality of GC are declining in many Asian countries, including South Korea, it still remains an important public health issue [2]. Therefore, the development of new anticancer drugs and effective therapeutic strategies for patients with GC is needed to increase the efficacy of treatment.

Topoisomerase (Topo) is a highly specialized nuclear enzyme involved in the correction of topological DNA errors during the elimination, replication, transcription, recombination, and chromosomal condensation of DNA [3,4]. Topo acts by sequentially breaking and recombining one or two strands of DNA, depending on the type of Topo involved in the process [5,6]. There are two types of Topo in humans: topoisomerase type I (Topo I) and topoisomerase type II (Topo II). Topo I breaks and recombines single strands of the double helix structure, while topo II cleaves and recombines both strands of DNA [7]. In fact, Topo activity, particularly inhibition of Topo I, is a key mechanism for a variety of anticancer agents. Inhibition of Topo Ι can lead to changes in DNA structure as well as DNA damage and can ultimately result in the induction of apoptosis [8].

Apoptosis is an essential process of programmed cell death in multicellular organisms. This cellular process prevents cancer by eliminating unwanted or unnecessary cells during development or by neutralizing cells that are potentially deleterious to DNA damage [9]. Apoptosis is initiated by various stresses, such as reactive oxygen species (ROS), DNA damage factors (e.g., radiation), heat shock, serum deprivation, viral infection, and hypoxia. ROS are considered a toxic product of cellular metabolism and can act as a signaling molecule that regulates many physiological processes [10]. Previous studies have shown that oxidative stress can lead to apoptosis through the extrinsic apoptotic receptor pathway as well as the endogenous mitochondrial apoptotic pathway [11,12].

Camptothecin is an alkaloid isolated from the stem wood of the Chinese tree, *Camptotheca acuminata*. This compound selectively inhibits the nuclear enzyme Topo I. However, because of its low solubility, numerous derivatives and analogues were synthesized. Among them, topotecan is approved by the U.S. FDA (Food and Drug Administration) for the treatment of ovarian and lung cancer. Another camptothecin derivative irinotecan is approved for the treatment of colorectal cancer. There are, however, certain clinical limitations of the camptothecin derivatives. These include: (1) spontaneous inactivation to the form of lactones in the blood, (2) resistance of cancer cells to camptothecins by overexpressing membrane transporters, and (3) dose-limiting side effects such as diarrhea and myelosuppression such as neutropenia [13,14]. To overcome these limitations, several laboratories are trying to develop non-camptothecin Topo I inhibitors.

**Figure 1:**
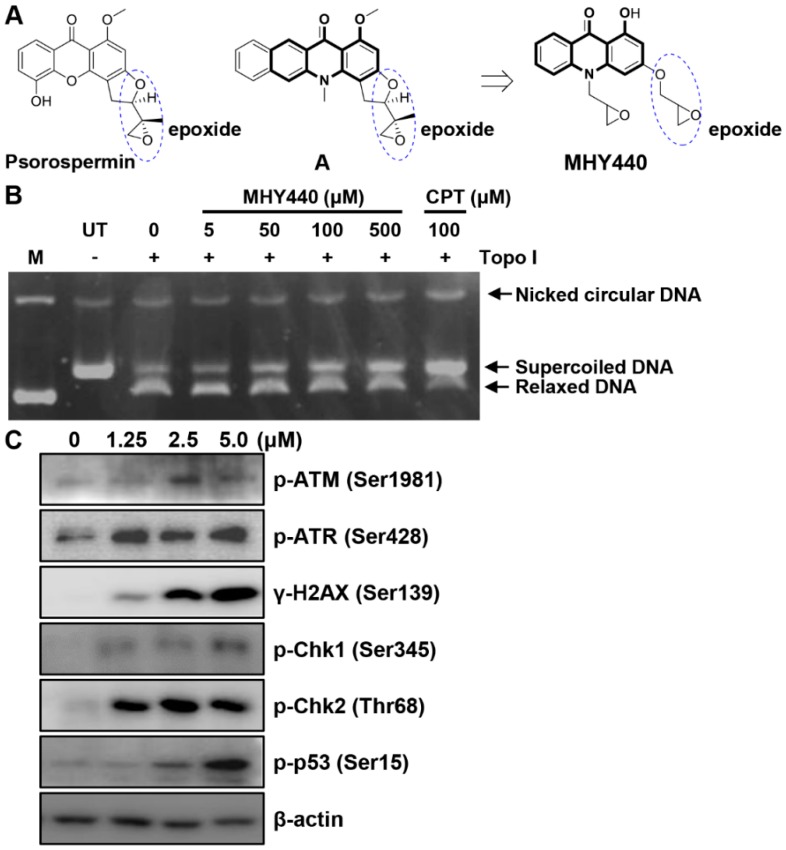
The chemical structure of the novel Topo I inhibitor, MHY440, and the effect of MHY440 on the DNA damage response in AGS cells. (**A**) The rationale for the design of MHY440 [1-hydroxy-3-((*R*/*S*)-oxiran-2-ylmethoxy)-10-((*R*/*S*)-oxiran-2-ylmethyl) acridin-9(10H)-one]. (**B**) Supercoiled substrate pHOT1 DNA (0.25 μg) was incubated with various concentrations of MHY440 (0, 5, 50, 100, and 500 μM) and Topo I at 37 °C for 30 min. The reaction product was separated on a 1% agarose gel containing EtBr. M, relaxed pHOT1 marker DNA; UT, supercoiled substrate pHOT1 DNA; CPT, camptothecin. (**C**) Western blot analysis of total cell lysates from cells treated with increasing concentrations of MHY440 for 24 h. The blots were probed with p-ATM (Ser1981), p-ATR (Ser428), γ-H2AX (Ser139), p-Chk1 (Ser345), p-Chk2 (Thr68), and p-p53 (Ser15). β-actin was used as a loading control. Representative results from three independent experiments are shown.

Psorospermin, a natural substance, showed topoisomerase II-induced DNA alkylation activity and compound A showed DNA alkylation activity (Figure 1A) [15,16]. Psorospermin and compound A each have a flat xanthone and benzo[*b*]acridinone template, and both compounds have an epoxy functional group in common at the similar position. For the discovery of a new anticancer agent, MHY440 with an epoxy group at the similar position and a flat acridinone template was designed and synthesized.

This study was conducted to characterize MHY440 [1-hydroxy-3-((*R*/*S*)-oxiran-2-ylmethoxy)-10-((*R*/*S*)-oxiran-2-ylmethyl) acridin-9(10H)-one] (Figure 1A) as a novel Topo I inhibitor, assess the cytotoxic effect of MHY440 on GC cells, and define the underlying molecular mechanism.

## 2. Results

### 2.1. Effects of MHY440 on Topo I and DNA Damage Signaling Pathway in AGS Cells

To confirm whether MHY440 inhibits Topo, a cell-free system was used. As shown in Figure 1B, MHY440 inhibited the activity of Topo I in a concentration-dependent manner. Camptothecin, a known Topo I inhibitor, was used as the positive control. Both camptothecin and MHY440 inhibited human Topo I and prevented the unwinding of the supercoiled DNA substrate. We confirmed that MHY440 is an inhibitor of Topo I; however, MHY440 did not demonstrate inhibition of Topo IIα (data not shown).

We next examined the expression of DNA damage-related proteins after treatment with MHY440. Ataxia telangiectasia mutated (ATM) is a well-known DNA damage sensor and regulator. After exposure to oxidative stress or DNA damage stresses, such as Topo I and II inhibitors, ATM kinase is activated by phosphorylation at Ser1981 and ataxia telangiectasia and Rad3-related (ATR) kinase is activated by phosphorylation at Ser428 [17]. The activation of these proteins by phosphorylation results in the phosphorylation of numerous downstream substrates, including Chk1, Chk2, p53, H2AX, etc., ultimately resulting in cell cycle arrest and apoptosis [18,19]. As shown in Figure 1C, the exposure of AGS cells to MHY440 markedly increased the protein levels of p-ATM, p-ATR, γ-H2AX, p-Chk1, p-Chk2, and p-p53 in a concentration-dependent manner. These results indicate that MHY440 is a novel Topo I inhibitor and induced a DNA damage signaling pathway in AGS cells.

### 2.2. Molecular Docking Simulation of Topo I with MHY440

**Figure 2:**
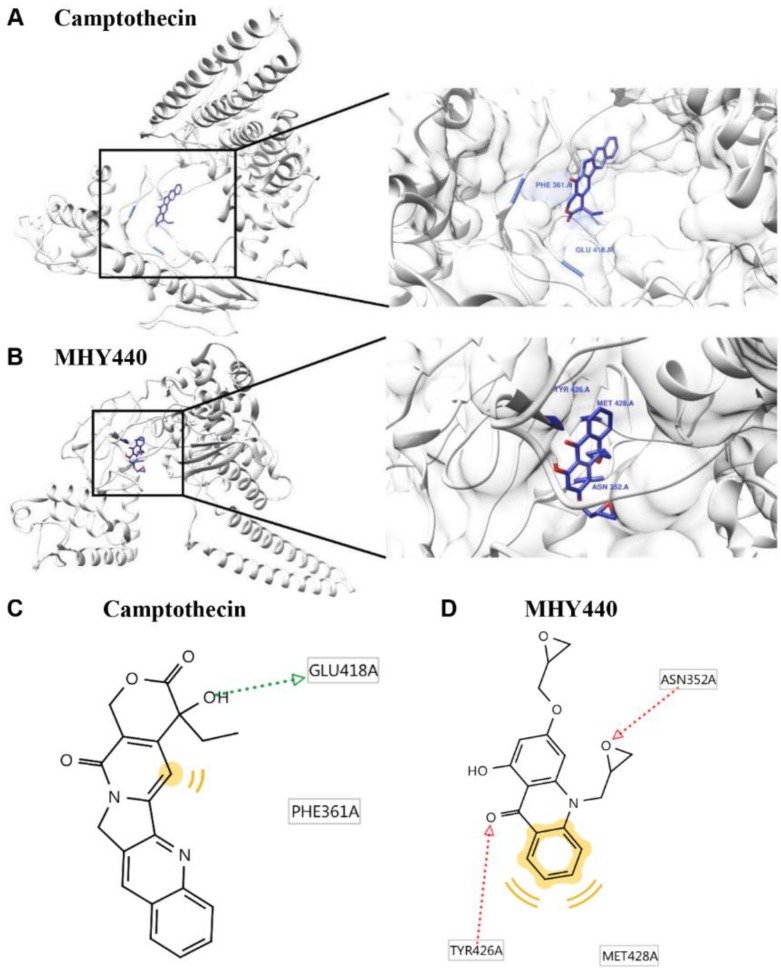
Computational structure prediction for docking simulation between Topo I and MHY440. (**A**) Predicted 3-D structure of Topo I. The black lines indicate the site of camptothecin. (**B**) Magnified image of the binding site of MHY440 in Topo I shows predicted binding affinity of MHY440 with Topo I. The binding sites of camptothecin (**C**) and MHY440 (**D**) with Topo I are shown. Pharmacophore results obtained using LigandScout 4.1.5 showing hydrogen-bonding (green and red arrows) and hydrophobic (yellow) interactions.

To further test the effect of MHY440 on Topo I, a protein-ligand docking simulation was performed using the AutoDock Vina and AutoDock 4 programs. The docking score between the ligand and the receptor can be expressed in terms of different energy sources including electrostatic energy, Van der Waals energy, and salvation energy. The predicted 3-D structure of Topo I, camptothecin (Figure 2A), and MHY440 (Figure 2B) are shown. The black lines indicate camptothecin and MHY440, respectively (Figure 2A,B). The docking simulation was effective with a considerable score. The binding energies of camptothecin and Topo I were −5.2 (Autodock Vina) and −5.73 (AutoDock 4) kcal/mol, respectively. The binding energies of MHY440 and Topo I were −4.4 (Autodock Vina) and −4.88 (AutoDock 4) kcal/mol, respectively (Table 1). Camptothecin interacted with the amino acid residue GLU418 with a single hydrogen bond, and MHY440 interacted with two active site amino acid residues ASN352 and TYR426 with two hydrogen bonds (Figure 2C,D and Table 1). The binding energy data in Table 1 indicated that camptothecin exhibits greater binding affinity than MHY440. However, MHY440 interacted by two hydrogen bonding with ASN352 and TYR426. In addition, TYR426 and MET428 residues were involved in hydrophobic interactions with MHY440. Camptothecin was the reported compound, interacted with two active-site amino acid residues, PHE361 and GLU418, with one hydrogen bond. PHE361 residue was involved in hydrophobic interaction with camptothecin. In general, it is known that the bond strength of hydrogen bonds is very strong, so the number of hydrogen bonds often means strength of bond strength. Thus, it can be predicted that MHY440 is more cohesive than camptothecin to Topo I.

### 2.3. Effects of MHY440 on the Growth of AGS Cells

**Figure 3:**
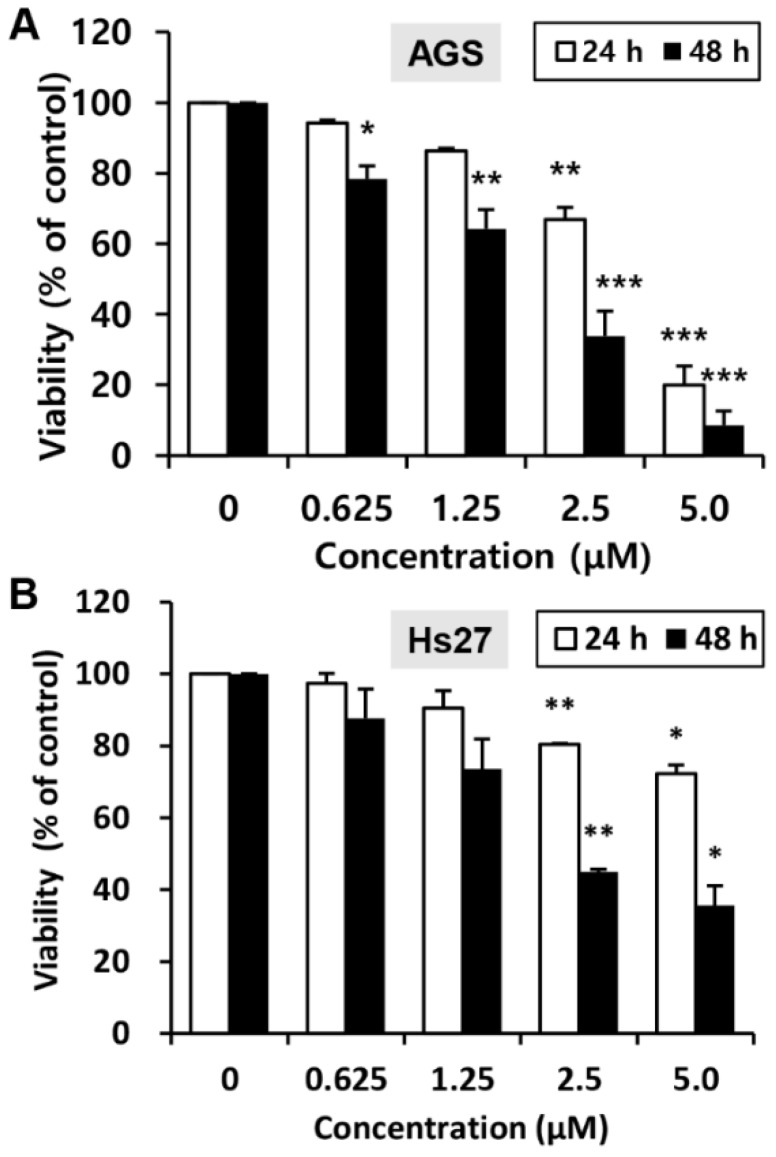
Effect of MHY440 on the viability of AGS human gastric cancer cells and Hs27 human foreskin fibroblast cells. (**A**) AGS cells were cultured with increasing concentrations of MHY440 for 24 and 48 h. The percentage of cell viability was then determined using the MTT assay. (**B**) Hs27 cells were treated with MHY440 for 24 and 48 h, and cell viability was determined by MTT assay. The results are expressed as a percentage of vehicle-treated controls ± standard deviation (SD) in three separate experiments. Significance was calculated by the Student’s *t*-test (* *p* < 0.05, ** *p* < 0.01, and *** *p* < 0.001 compared with vehicle-treated cells).

To investigate the effects of MHY440 on the viability of the human GC cell line (AGS) and the human foreskin fibroblast cell line (Hs27), the 3-(4,5-dimethylthiazol-2-yl)-2,5-diphenyl tetrazolium bromide (MTT) assay was performed. Cell viability was reduced with MHY440 treatment in a concentration- and time-dependent manner. As shown in Figure 3A, in AGS cells, the half-maximal inhibitory concentration (IC_50_) of MHY440 at 24 and 48 h was 3.40 μM and 1.83 μM, respectively. However, MHY440 exhibited decreased cytotoxic effects on Hs27 cells (Figure 3B), suggesting MHY440 exerts its inhibitory effects specifically on the proliferation of cancer cells. Therefore, only AGS cells were used for further experiments.

### 2.4. Effects of MHY440 on the Cell Cycle in AGS Cells

**Figure 4:**
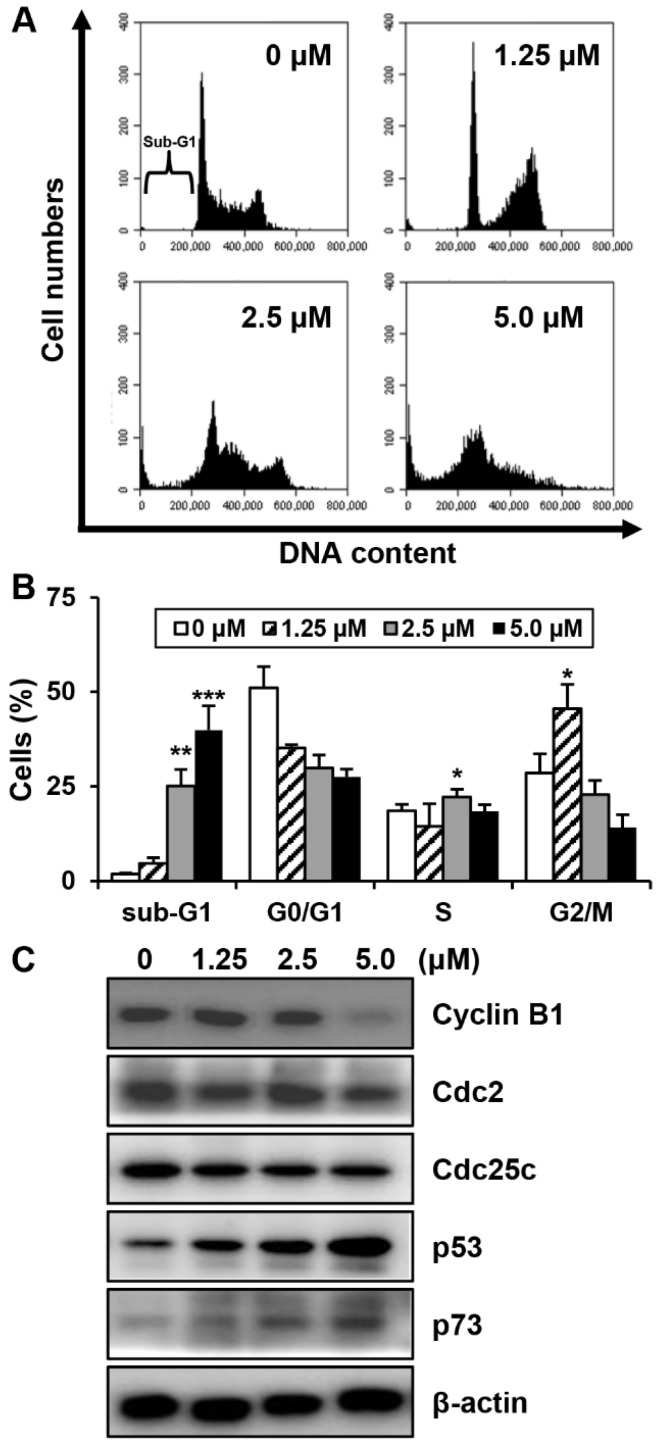
The effect of MHY440 on cell cycle regulation in AGS cells. (**A**) Cells were treated with MHY440 at indicated concentrations for 24 h, stained with propidium iodide (PI), and then subjected to flow cytometry analysis to determine their distribution at each phase of the cell cycle. Representative results from three independent experiments are shown. (**B**) Results are expressed as means ± SD of four independent experiments. Significance was determined using Student’s *t*-test (* *p* < 0.05, ** *p* < 0.01, and *** *p* < 0.001 compared with vehicle-treated cells). (**C**) After MHY440 treatment for 24 h, cells were subjected to western blot analysis for the following proteins: cyclin B1, Cdc2, Cdc25c, p53, and p73. β-actin was used as a protein loading control. Representative results from three independent experiments are shown.

To investigate whether MHY440 affects cell cycle distribution, AGS cells were treated with various concentrations of MHY440 for 24 h and then analyzed for cell cycle progression using flow cytometry. As shown in Figure 4A, MHY440 exposure resulted in an accumulation of cells at G2/M phase. Flow cell analysis demonstrated that 45.58% of cells cultured with 1.25 μM MHY440 were in G2/M phase compared to 28.54% of control cells. In addition, the sub-G1 population increased from 1.88% in the control group to 39.87% in cells treated with 5.0 μM MHY440 (Figure 4B). Next, we examined whether MHY440 regulates the expression of G2/M cell cycle regulators. Cells were treated with various concentrations of MHY440 for 24 h and the level of G2/M cell cycle regulating proteins were examined using western blot analysis. As shown in Figure 4C, MHY440 treatment markedly decreased cyclin B1 in a concentration-dependent manner in AGS cells; Cdc2 and Cdc25c proteins were also slightly decreased. The transcription factor p53 is induced by a number of stress signals. Cell cycle arrest and apoptosis are the most prominent results of p53 activation [20]. In addition, p73 is a protein associated with p53, and it is considered a tumor suppressor because it is structurally similar to p53. It is involved in cell cycle regulation and induction of apoptosis [21]. Therefore, we examined the expression of p53 and p73 in AGS cells treated with MHY440. Our results show that MHY440 treatment increased the expression of both p53 and p73 in a concentration-dependent manner in AGS cells (Figure 4C). In summary, these results indicate that MHY440 induced cell cycle arrest by controlling the expression of key proteins involved in the regulation of G2/M phase in AGS cells.

### 2.5. Effects of MHY440 on the Induction of Apoptosis in AGS Cells

**Figure 5:**
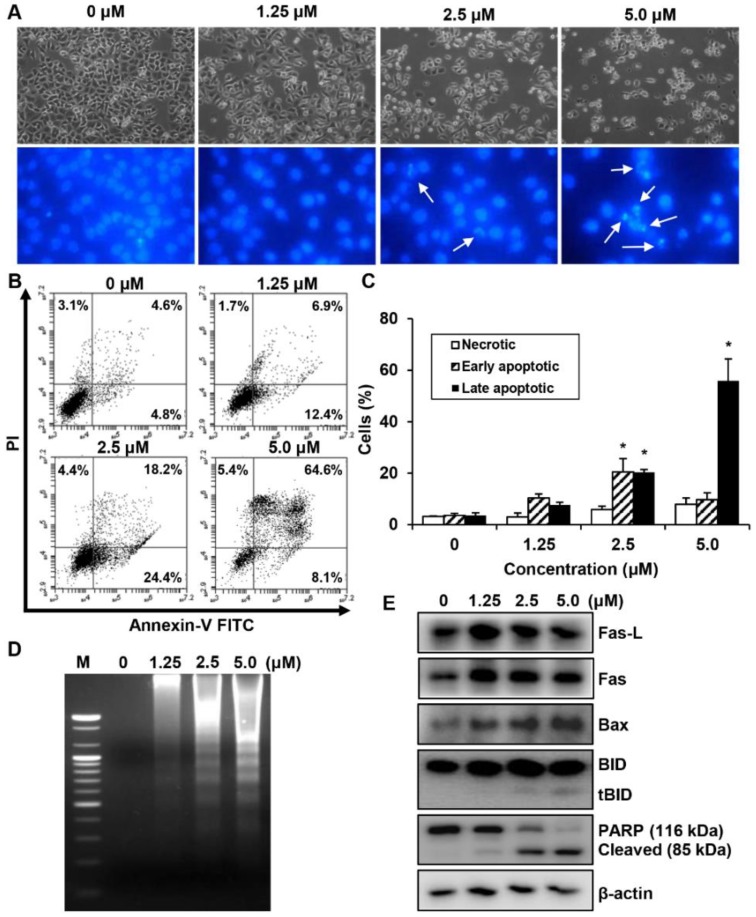
The effect of MHY440 on apoptosis in AGS cells. (**A**) Morphological changes of MHY440-treated cells. Nuclei of AGS cells stained with Hoechst 33342 to visualize DNA. The stained cells and nuclei were then observed with a fluorescence microscope (original magnification, 400×). Arrows indicate apoptotic cells. (**B**) The effect of MHY440 on cell death was determined via Annexin V-FITC/PI analysis using flow cytometry. (**C**) Results are expressed as the means ± SD of three independent experiments. Significance was determined using Student’s *t*-test (* *p* < 0.05 compared with vehicle-treated cells). (**D**) Representative results of DNA analysis from three independent experiments are shown. M, Marker. (**E**) Western blot analysis of total cell lysates of cells treated with increasing concentrations of MHY440 for 24 h. The blots were probed with antibodies against Fas-L, Fas, Bax, BID, and PARP. β-actin was used as a loading control. Representative results from three independent experiments are shown.

We investigated whether the MHY440-dependent growth inhibition in AGS cells is mediated by apoptosis via analyzing the features of nuclear morphological changes. AGS cells treated with MHY440 displayed cell shrinkage and rounding as well as a decrease in cell number in a concentration-dependent manner compared with the untreated control group. Hoechst 33342 staining confirmed the induction of apoptosis in AGS cells treated with MHY440 for 24 h. MHY440-treated cells showed nuclear fragmentation, which is characteristic of chromatin condensation and apoptosis, whereas control cells showed normal circular morphology of the nucleus (Figure 5A). To confirm that MHY440-induced cell death was indeed apoptosis, we performed flow cytometry using Annexin V and PI staining. As shown in Figure 5B, the ratio of late apoptotic cells (upper right quadrant, Annexin V/PI positive) increased from 4.6% to 64.6% after 24 h of exposure to 5.0 μM MHY440. The results of flow cytometry also indicated that MHY440-induced apoptosis was concentration-dependent (Figure 5C). Treatment of AGS cells with MHY440 for 24 h resulted in a concentration-dependent internucleosomal DNA fragmentation (Figure 5D). To investigate the molecular mechanism of apoptotic cell death by MHY440 treatment, western blot analysis was conducted with the antibodies for apoptotic marker proteins. As shown in Figure 5E, MHY440 upregulated the death receptor Fas and its ligand Fas-L in a concentration-dependent manner. In addition, the expression of the pro-apoptotic protein Bax by MHY440 treatment was increased compared to the control groups. Furthermore, the levels of total BID expression were decreased with MHY440 treatment, but truncated Bid (tBid) expression progressively increased depending on the concentration of MHY440 treatment. tBid localizes at the mitochondrial membrane and can stimulate the release of cytochrome *c* and promote the progression of apoptosis [11,12]. MHY440 treatment also caused proteolytic degradation of PARP, a molecular marker of apoptosis. Cleavage of PARP was evident by the appearance of an 85 kDa fragment (uncleaved PARP protein is 116 kDa) observed by western blot analysis in AGS cells treated with either 2.5 µM or 5.0 µM of MHY440. These results suggest that MHY440 induced apoptosis in AGS cells.

### 2.6. Effects of MHY440 on the Mitochondrial Membrane Potential (MMP)

**Figure 6:**
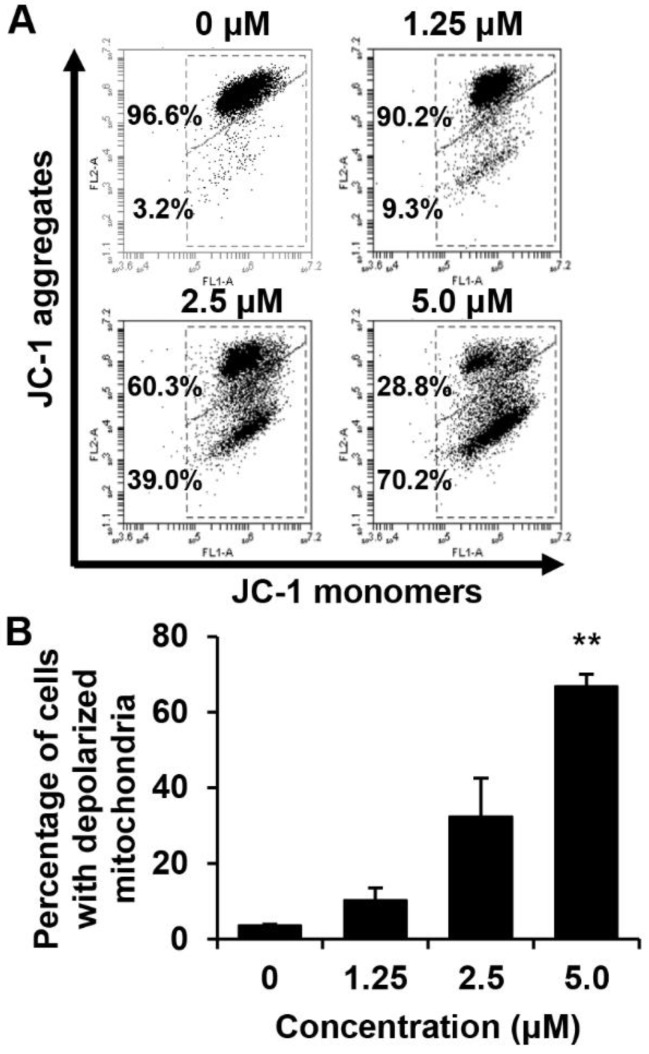
The effect of MHY440 on the mitochondrial membrane potential (ΔΨm) in AGS cells. (**A**) To analyze ΔΨm, AGS cells were incubated with the indicated concentrations of MHY440 for 24 h, stained with JC-1 dye, and analyzed using flow cytometry. The data shown represent three independent experiments with similar results. (**B**) Quantitative data are provided by means of green fluorescence (depolarized ΔΨm) in the monomeric form of JC-1. The results are expressed as means ± SD of three individual experiments. Significance was determined using Student’s *t*-test (** *p* < 0.01 compared with vehicle-treated cells).

We determined whether the death of these cells is related to the disruption of MMP. The effect of MHY440 on the MMP in AGS cells was determined using flow cytometry after staining with 5,5′,6,6′-tetrachloro-1,1′,3,3′-tetra-ethylbenzimidazolylcarbocyanine iodide (JC-1), (a lipophilic cationic dye selectively entering mitochondria). The frequency of cells with JC-1 monomers, which are predominant in regions with low MMP (lower right panel of fluorescence cytogram), increased in MHY440-treated AGS cells, suggesting that MHY440 destroyed the integrity of the mitochondria, as measured by the concentration-dependent loss of MMP (Figure 6A). The average populations of AGS cells with damaged membrane potentials from three individual experiments were 3.6%, 10.3%, 32.4%, and 66.8% at 0, 1.25, 2.5, and 5.0 μM MHY440 concentration, respectively (Figure 6B). Overall, these results indicate that mitochondrial dysfunction may also contribute to MHY440-induced apoptosis in AGS cells.

### 2.7. Effects of MHY440 on the Caspase Activation

**Figure 7:**
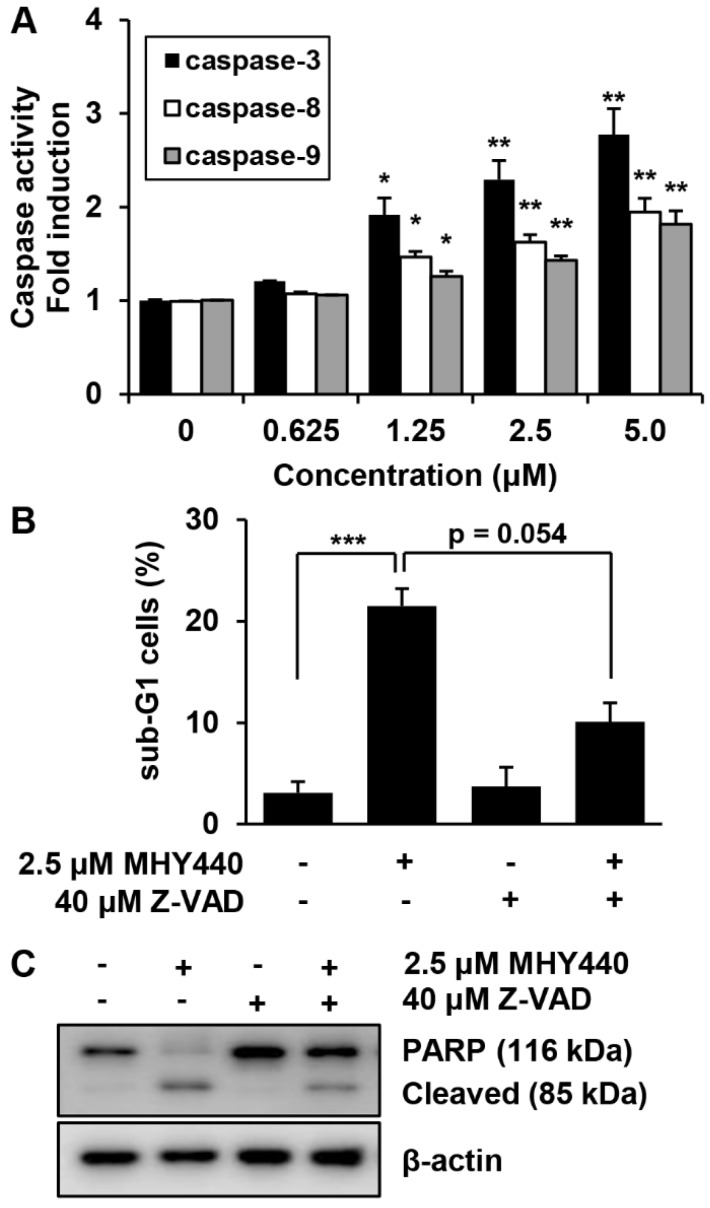
The effect of caspases on MHY440-induced apoptosis in AGS cells. (**A**) MHY440-treated cell lysates were assayed for caspase-3, -8, and -9 activities using DEVD-pNA, IETD-pNA and LEHD-pNA substrates, respectively. The emitted fluorescent products were measured. Data are expressed as the means ± SD of triplicate samples. The results represent one of three independent experiments. (**B**) Cells were pretreated with 40 μM Z-VAD-FMK for 30 min and then treated with 2.5 μM MHY440 for 24 h. Cells were stained with PI and analyzed using flow cytometry. The results are expressed as means ± SD of three individual experiments. Significance was determined using Student’s *t*-test (* *p* < 0.05, ** *p* < 0.01 and *** *p* < 0.001 compared with vehicle-treated cells). (**C**) Total cell lysates were prepared and immunoblotted for PARP. β-actin was used as a loading control. Representative results from three independent experiments are shown.

Pro-caspases are precursors of caspases, which are the executors of the apoptotic process [22]. However, a decrease in the level of a pro-caspase may not accurately reflect the activation of caspase. We, therefore, evaluated the effect of MHY440 on caspase activation using specific substrates. The histograms in Figure 7A show that caspase-3 activation was increased almost three-fold in AGS cells treated with 5.0 μM MHY440, while caspase-8 and caspase-9 did not increase more than two-fold (Figure 7A). To identify the relevance of caspase activation in MHY440-induced apoptosis, AGS cells were cultured in the presence and absence of the broad-spectrum caspase inhibitor Z-VAD-FMK and analyzed using flow cytometry and western blot analysis. As shown in Figure 7B, pretreatment of cells with Z-VAD-FMK partially decreased the accumulation of sub-G1 fractions induced by MHY440. To further demonstrate this result, western blot analysis for PARP cleavage was conducted using the same experimental conditions. Consistent with the cell death measured by flow cytometry, western blot analysis of PARP showed that pretreatment with Z-VAD-FMK significantly inhibited the cleavage of MHY440-induced PARP (Figure 7C). These results suggest that activation of caspases contributed to the MHY440-induced apoptosis in AGS cells.

### 2.8. Effects of MHY440 on the ROS Generation

**Figure 8:**
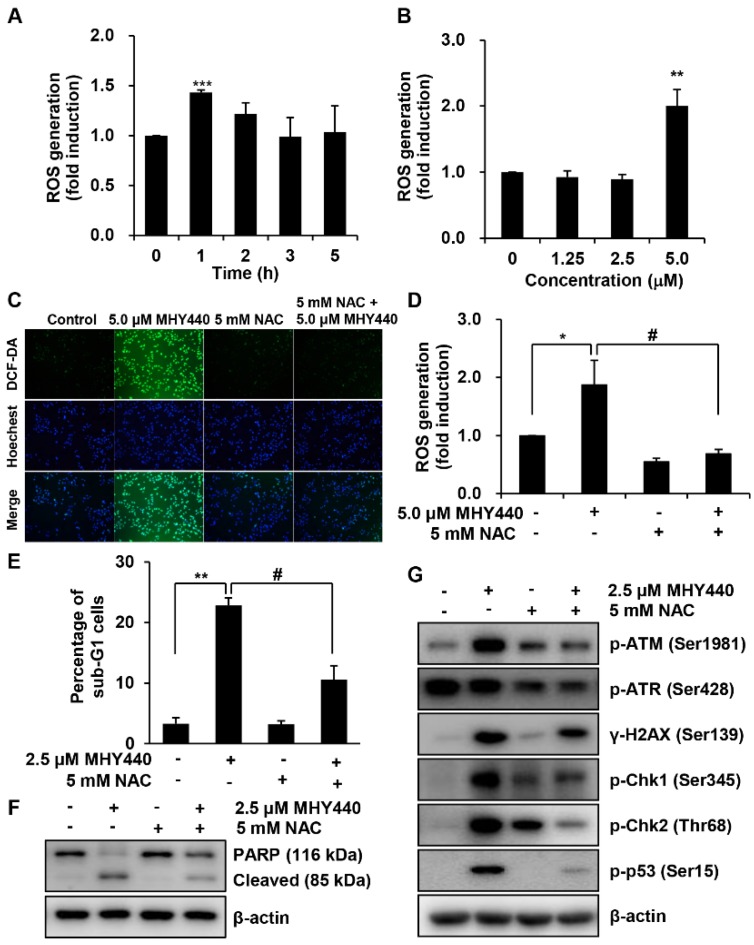
The role of ROS on MHY440-induced apoptosis in AGS cells. (**A**) Cells were treated with 5.0 μM MHY440 for several different times and stained with DCF-DA. Intracellular ROS levels were measured using flow cytometry. Data are means ± SD of three separate experiments. Significance was determined using Student’s *t*-test (*** *p* < 0.001 compared with vehicle-treated cells). (**B**) Cells were treated at various concentrations for 1 h. Data are expressed as the means ± SD of three separate experiments. Significance was determined using Student’s *t*-test (** *p* < 0.01 compared with vehicle-treated cells). (**C**) Cells were pretreated with or without 5 mM NAC for 1 h and then treated with 5.0 μM MHY440 for 1 h. Intracellular ROS levels were measured using fluorescence microscopy. Representative results from three independent experiments are shown. (**D**) Cells were treated with 5.0 μM MHY440 for 1 h after pretreatment with or without 5 mM NAC for 1 h. Data are means ± SD of three separate experiments. Significance was determined using Student’s *t*-test (* *p* < 0.05 compared with vehicle-treated cells; ^#^ *p* < 0.05 compared with 5.0 μM MHY440-treated cells). (**E**) The expression of apoptosis in cells pretreated with 5 mM NAC and 2.5 μM MHY440 was determined using PI staining and flow cytometry analysis. Data are means ± SD of three separate experiments. Significance was determined using Student’s *t*-test (** *p* < 0.01 compared with vehicle-treated cells; ^#^ *p* < 0.05 compared with 5.0 μM MHY440-treated cells). (**F**) Total cell lysates of cells treated with or without 2.5 μM MHY440 after pretreatment with or without 5 mM NAC were analyzed using western blot analysis for the expression level of PARP. β-actin was used as a loading control. Representative results from three independent experiments are shown. (**G**) Total cell lysates from cells treated with 2.5 μM MHY440 alone or pretreated with 5.0 mM NAC for 24 h were analyzed using western blot analysis for the following antibodies: p-ATM (Ser1981), p-ATR (Ser428), γ-H2AX (Ser139), p-Chk1 (Ser345), p-Chk2 (Thr68), and p-p53 (Ser15). β-actin was used as a loading control. Representative results from three independent experiments are shown.

We examined the production of ROS in AGS cells after treatment with MHY440 and determined whether this was the mechanism for the induction of apoptosis. Intracellular ROS levels were quantified using the fluorescent probe 2′,7′-dichlorofluorescein diacetate (DCF-DA). AGS cells were treated with 5.0 μM MHY440 for various times. As shown in Figure 8A, the maximum intracellular level of ROS was at 1 h after exposure to 5.0 μM MHY440. When AGS cells were treated for 1 h with increasing concentrations of MHY440, ROS generation was most abundant in 5.0 μM MHY440 treatment (Figure 8B). AGS cells were then pretreated with N-acetyl-L-cysteine (NAC), a well-known ROS scavenger, and then exposed to 5.0 μM MHY440 for 1 h. At the end of the incubation period, AGS cells were analyzed using fluorescence microscopy and the levels of intracellular ROS were measured. As shown in Figure 8C, when AGS cells were treated with 5.0 μM MHY440, ROS was generated, and a green color appeared in the cells. However, pretreatment of cells with NAC considerably blocked the generation of ROS in MHY440-treated AGS cells, as evidenced by a decrease in green color (Figure 8C,D).

To further confirm the relationship between ROS generation and apoptosis, the effect of NAC was evaluated in cells treated with MHY440. As shown in Figure 8E, after exposure to with MHY440 with or without NAC pretreatment, the presence of cells with sub-G1 DNA content was assessed using flow cytometry to quantify the onset of apoptosis. Cells pretreated with NAC significantly inhibited apoptosis in MHY440-treated cells. Consistent with these observations, sequestration of ROS by NAC effectively inhibited MHY440-induced PARP cleavage in AGS cells (Figure 8F). In addition, to investigate the effect of ROS generation on the DNA damage response, we examined the effects that treatment of MHY440 with or without NAC had on the expression of DNA damage response proteins. We found that the inhibition of ROS by NAC effectively down-regulated the levels of MHY440-induced DNA damage response proteins, including p-ATM, p-ATR, γ-H2AX, p-Chk1, p-Chk2, and p-p53, all of which were increased after MHY440 treatment alone (Figure 8G). These results demonstrate that ROS generation played an important role in the MHY440-mediated apoptotic pathways as well as the DNA damage response pathways in AGS cells.

## 3. Discussion

DNA Topo Ι controls the topological state of DNA in many cell processes, including DNA replication and transcription [8]. Compounds that inhibit Topo I activity have been widely used as anticancer agents because of their ability to block DNA damage, trigger cell cycle arrest, and subsequently initiate apoptosis [23]. FDA-approved Topo I inhibitors camptothecin derivatives topotecan and irinotecan are currently used in the treatment of ovarian and colon cancer, respectively [24]. Based on these reports, we examined the effect of MHY440 on HCT116 human colon cancer cells and AGS human gastric cancer cells. After 24 h of MHY440 treatment, the IC_50_ of HCT116 cells and AGS cells was 5.24 μM and 3.40 μM, respectively. Based on these preliminary results, whole experiments were conducted using AGS human gastric cancer cell line.

Induction of DNA damage is a key mechanism of Topo inhibitors [25]. Suppression of Topo activity and induction of DNA damage stimulates DNA repair enzymes [26]. DNA damage pathways include damage sensors, signal transducers, and effectors. DNA damage causes activation of DNA damage response elements, such as ATM and ATR. Activation of ATR is generally associated with single-stranded DNA damage or arrest of DNA replication forks, whereas ATM activation is associated with the initiation of signaling pathways involved with double-strand DNA breaks [26]. During the inhibition of Topo activity, activated ATM and ATR directly influence the downstream proteins BRCA1, H2AX, Chk1, and Chk2 through either direct or sequential steps, resulting in the inhibition of downstream factors involved in cell cycle progression and cell survival [27]. Phosphorylated H2AX and BRCA1 are involved in DNA repair and the activation of other repair factors, but phosphorylated Chk1 and Chk2 activate cell cycle arrest and apoptosis-related factors [28].

It is well known that the progression of the cell cycle is tightly regulated by the interaction between the activators of the cell cycle and inhibitors of the cell cycle. The progression of the eukaryotic cell cycle is controlled by the coordinated activity of the Cdk-cyclin complex [29]. G2/M transitions are primarily dependent on cyclin B1/Cdk1 activity. The activity of cyclin B1/Cdk1 can be activated by Cdc25c or inhibited by p53, p21^Waf1/Cip1^, and p27^Kip1^ [30]. Cdc25c is a key protein controlling cell cycle G2/M transition and is an essential component of the checkpoint pathway that can be activated in response to DNA damage. Activation of ATM by DNA damage mediates the induction of Chk1 and Chk2, inhibition and degradation of Cdc25c, and activation of Cdk1 via Cdc25c, all of which result in cell cycle blockade at G2/M [31]. In our study, exposure of AGS cells to MHY440 significantly induced the activation of ATM, ATR, Chk1, and Chk2 via phosphorylation. Activation of the ATM/ATR and Chk1/2 signaling axes inhibited Cdc25c, which in turn, inhibited cyclin B1/Cdk1 kinase activity and induced cell cycle arrest in the G2/M phase (Figure 1C; Figure 4B,C).

The tumor suppressor protein, p53, is an important component of the cell machinery, which regulates a variety of signaling pathways, including carcinogenesis, cell cycle, apoptosis, and DNA damage responses under a variety of conditions. In response to Topo suppression or chemically-induced DNA damage, activated ATM or Chk2 directly activates p53 via phosphorylation, which inhibits its interaction with the negative regulator murine double minute2 (MDM2) [32]. Activated p53 induces Bax expression, which leads to an imbalance in the Bax/Bcl-2 ratio, resulting in the release of cytochrome *c* from the mitochondria, disruption of the mitochondrial membrane potential, and the induction of apoptosis. In our study, MHY440-treated AGS cells showed increased expression of p53 and Bax in addition to increased proteolysis of the BID protein (Figure 4C and Figure 5E). MHY440 also caused loss of mitochondrial membrane potential in AGS cells (Figure 6A,B).

In biological systems, ROS are constantly generated and removed. ROS also play an important role in both homeostasis and disease. The excessive production of ROS in the mitochondria is known to play an important role in the regulation of apoptosis [33]. The downregulation of survivin, a member of inhibitor of apoptosis and an antagonist of apoptosis, was associated with ROS production in cancer cell apoptosis [34,35,36]. Some anticancer agents, such as cisplatin, doxorubicin, mitomycin C, and etoposide, are at least partially effective through the induction of ROS [37]. Oxidative stress induced by ROS can damage cellular components, including DNA and proteins [38]. The continued failure of cells to repair DNA lesions with the appropriate repair mechanisms can eventually translate into double-strand DNA breaks, which ultimately lead to cell cycle arrest and cell death. Several studies have shown that ROS can affect cell cycle progression and cell death by activating intracellular signaling pathways sensitive to various oxidative stresses, such as ATM/ATR, Chk1/2, and c-Jun N-terminal kinases (JNK) [39].

In conclusion, MHY440, as a novel Topo Ι inhibitor, inhibited the growth of AGS cells by inducing a DNA damage response, arresting cell cycle at G2/M phase, and initiating apoptosis through the activation of a caspase cascade and ROS generation. Overall, our results demonstrate that MHY440 has the potential to be used as a therapeutic agent for treating GC.

## 4. Materials and Methods

### 4.1. Chemicals and Reagents

The simplified code name and structure of MHY440 used in this study are shown in Figure 1A. This compound was provided by Professor Hyung Ryong Moon (Pusan National University, Busan, Korea). MHY440 was synthesized via condensation of methyl anthranilate with phloroglucinol in the presence of *p*-toluenesulfonic acid followed by alkylation with epichlorohydrin in the presence of potassium carbonate as shown in Supplementary material (Scheme 1). Compound **1** was obtained in a yield of 58% and alkylation of compound **1** with epichlorohydrin afforded the desired product MHY440 and a monoalkylated product, compound **2**, at a yield of 16 and 20%, respectively. MHY440 was dissolved in sterile dimethyl sulfoxide (DMSO) to make a 10 mM stock solution and stored at −20 °C until use. Subsequent dilutions were performed in RPMI-1640 (GE Healthcare Life Sciences, Logan, UT, USA). The maximum concentration of DMSO did not exceed 0.1% (*v*/*v*), which did not affect cell growth. DMSO and 3-(4,5-dimethylthiazol-2-yl)-2,5-diphenyl tetrazolium bromide (MTT) were obtained from Amresco Inc. (Solon, OH, USA). Camptothecin, propidium iodide (PI), *N*-acetyl-l-cysteine (NAC), and the monoclonal antibody against β-actin were purchased from Sigma-Aldrich (St. Louis, MO, USA). Antibodies specific for phosphorylated (p-) ataxia telangiectasia-mutated kinase (ATM, Ser1981), p-ataxia telangiectasia and rad3-related kinase (ATR, Ser428), γ-H2AX (Ser139), p-checkpoint kinase 1 (Chk1, Ser345), p-Chk2 (Thr68), p-p53 (Ser15), and BH3-interacting domain death agonist (BID) were purchased from Cell Signaling Technology (Danvers, MA, USA). Cyclin B1, cell division cycle protein 2 (Cdc2), Cdc25c, p53, p73, Fas-L, Fas, Bcl-2-associated X protein (Bax), poly(ADP-ribose) polymerases (PARP), and Z-VAD-FMK were obtained from Santa Cruz Biotechnology, Inc. (Dallas, TX, USA).

### 4.2. Determination of Topo I Activity

A commercially available Topo Ι drug screening kit (TopoGEN, Inc., Buena Vista, CO, USA) was used in this study, per the manufacturer’s instructions. Briefly, supercoiled substrate pHOT1 DNA substrate (0.25 μg) was incubated with Topo Ι reaction buffer (2 μL), MHY440, and four units of human DNA Topo Ι at 37 °C for 30 min. After the incubation, 10% sodium dodecyl sulfate (2 μL) was added, and then the reaction was terminated via decomposition with proteinase K (50 μg/mL) at 37 °C for 15 min. The DNA was resolved on a 1% agarose gel in a gel electrophoresis system for 90 min. Ethidium bromide (EtBr) was added to the gel to aid in visualizing the DNA. The gel images were determined via BioSpectrum AC imaging system (UVP, Upland, CA, USA).

### 4.3. Docking Simulation of Topo I and MHY440

Because of automated docking capability, AutoDock Vina and AutoDock 4 were used for the in silico protein–ligand docking simulation. The 3-D structure of Topo I was used in the crystal structure of human Topo I (PDB ID: 1K4T). As a docking pocket, predefined binding site of Topo I was used [40]. Docking simulations were performed between Topo I and MHY440 or camptothecin. To prepare compounds for docking simulation, (1) 2-D structures were converted into 3-D structures, (2) charges were calculated, and (3) hydrogen atoms were added using the ChemSketch program (http://www.acdlabs.com/resources/freeware/chemsketch). The prediction of possible hydrogen bonding residues between compounds and Topo I and generation of pharmacophores were analyzed with LigandScout 4.1.5 program (Inte:Ligand, Vienna, Austria).

### 4.4. Cell Culture and Cell Viability Analysis

The human GC cell line (AGS) and the human foreskin fibroblast cell line (Hs27) were purchased from the American Type Culture Collection (Manassas, VA, USA) and grown in RPMI-1640 supplemented with 10% fetal bovine serum (GE Healthcare Life Sciences), 100 U/mL penicillin, and 100 μg/streptomycin (GE Healthcare Life Sciences) and incubated at 37 °C with 5% CO_2_. Cell viability was measured using an MTT assay. For MTT analysis, cells were incubated on 24-well culture plates and cultured for 24 or 48 h and then treated with or without various reagents at the indicated concentrations of MHY440. Cells were incubated with 0.5 mg/mL MTT in the dark at 37 °C for 2 h. The formazan produced by live cells were dissolved in DMSO, and the absorbance at 540 nm was monitored with a multi-wall reader (Thermo Fisher Scientific, Waltham, MA, USA).

### 4.5. Cell Cycle Analysis

Cells were treated for 24 h at the appropriate conditions and then were treated with trypsin. The cells were then washed one time with cold phosphate-buffered saline (PBS) and then stored in 70% ethanol overnight at −20 °C. Fixed cells were pelleted and stained in cold PI solution (50 μg/mL in PBS) in the dark for 30 min at room temperature. The stained DNA contents of the cells were then analyzed using flow cytometry on a Accuri C6 flow cytometer (BD Biosciences, Franklin Lakes, NJ, USA).

### 4.6. Western Blot Analysis

Cells were harvested, lysed, and an equivalent amount of protein was subjected to sodium dodecyl sulfate-polyacrylamide gel electrophoresis and then transferred onto polyvinylidene fluoride membranes for immunoblotting. The membranes were blocked with 5% nonfat dry milk in Tris-buffered saline for 1 h with a Tween-20 buffer (TBS-T; 20 mM Tris, 100 mM NaCl, pH 7.5, and 0.1% Tween-20) at room temperature. Membranes were then incubated with primary antibodies overnight at 4 °C. The membranes were washed with a TBS-T buffer and incubated with horseradish peroxidase-conjugated secondary antibody (Santa Cruz Biotechnology) for 1 h. The membranes were washed with TBS-T buffer. Antigen-antibody complexes were detected using an enhanced chemiluminescence detection system (GE Healthcare, Chicago, IL, USA).

### 4.7. Nuclear Staining with Hoechst 33342

Cells were stained with 4 μg/mL Hoechst 33342 (Life Technologies Corp., Grand Island, NY, USA) at 37 °C for 10 min. Cells were examined by a Nikon Eclipse TE 2000-U microscope (Nikon, Tokyo, Japan).

### 4.8. Annexin V Staining

The proportion of cells that actively progressed to apoptosis was quantitatively determined using an Annexin V-FITC apoptosis detection kit (BD Biosciences). Cells were treated for 24 h at the appropriate conditions and then harvested, treated with trypsin, washed once in cold PBS, and resuspended in 1X Binding Buffer. The aggregated cells were stained in the dark for 15 min at room temperature in PI and Annexin V-FITC solution. The stained cells were analyzed by an Accuri C6 flow cytometer.

### 4.9. DNA Fragmentation Assay

Cells were lysed on ice for 30 min in a buffer containing 5 mM Tris-HCl (pH 7.5), 5 mM EDTA, and 0.5% Triton X-100. The lysate was vortexed and then centrifuged at 27,000× *g* for 20 min. The fragmented DNA in the supernatant was RNase-treated and then treated with proteinase K to degrade any exogenous proteins. The DNA was then extracted with a phenol/chloroform/isoamyl alcohol mixture (25:24:1, *v*/*v*/*v*) and precipitated with isopropanol. DNA was separated from 1.6% agarose gel, stained with 0.1 μg/mL EtBr, and visualized with a UV light source.

### 4.10. Measurement of Mitochondrial Membrane Potential (MMP, ΔΨm)

MMP was measured using a flow cytometer and a lipophilic cationic dye, 5,5′,6,6′-tetrachloro-1,1′,3,3′-tetra-ethylbenzimidazolylcarbocyanine iodide (JC-1; Calbiochem, San Diego, CA, USA). JC-1 is a dye that stains the mitochondria of living cells in a membrane potential-dependent manner. Cells were treated with various concentrations of MHY440, harvested, and washed with cold PBS. Cells were stained with 10 μM JC-1 for 20 min at 37 °C in the dark. Cells were then washed with cold PBS and analyzed using an Accuri C6 flow cytometer.

### 4.11. Measurement of Caspase Activity

Cells were harvested, washed with cold PBS, and incubated with a lysis buffer (R&D Systems, Inc., Minneapolis, MN, USA) for 10 min on ice. The lysed cells were centrifuged at 10,000× *g* for 1 min, and 100 μg of protein was added to the reaction mixture containing 2× reaction buffer and substrates of colorimetric tetrapeptides, including DEVD-pNA for caspase-3, IETD-pNA for caspase-8, and LEHD-pNA for caspase-9. The reaction mixture was incubated at 37 °C for 2 h, and then enzymatic release of p-nitroaniline was quantitated at 405 nm using a multi-wall reader (Thermo Fisher Scientific).

### 4.12. Measurement of Intracellular ROS Accumulation

The intracellular accumulation of ROS was monitored using the fluorescent probe 2′,7′-dichlorofluorescin diacetate (DCF-DA). A solution of 10 μM DCF-DA was added to the cells. After incubation at 37 °C for 30 min, the intracellular accumulation of ROS was determined by a Nikon Eclipse TE 2000-U microscope set at 488 nm for excitation and 530 nm for emission. Alternatively, cells were rinsed with PBS, treated with trypsin, washed with PBS, and then analyzed by an Accuri C6 flow cytometer.

### 4.13. Statistical Analysis

Data are presented as means ± standard deviations (SD) of three separate experiments and analyzed via Student’s t-test. The mean was considered significantly different if * *p* < 0.05, ** *p* < 0.01, and *** *p* < 0.001.

## Figures and Tables

**Table 1:** Topo I inhibitory activity of MHY440.

Compounds	Binding Energy (kcal/mol) ^a^		No. of H-Bond ^b^	H-Bond Interacting Residues ^b^	Van der Waals Bond Interaction Residues ^b^
	AutoDock Vina	AutoDock 4			
Camptothecin	−5.2	−5.73	1	GLU418	PHE361
MHY440	−4.4	−4.88	2	ASN352, TYR426	TYR426, MET428

^a^ The binding energy represents the binding affinity and capacity for the active site of the Topo I enzyme. ^b^ The number of hydrogen bonds and all amino acid residues of the enzyme-inhibitor complex were determined using the AutoDock Vina and AutoDock 4 programs.

## Supplementary Materials

The following are available online.
