# Supplementary material for: MHY440, a Novel Topoisomerase Ι Inhibitor, Induces Cell Cycle Arrest and Apoptosis via a ROS-Dependent DNA Damage Signaling Pathway in AGS Human Gastric Cancer Cells
Source: Molecules. 2018 Dec 28;24(1):96. doi: 10.3390/molecules24010096 (PMC6337620; doi:10.3390/molecules24010096)
Supplement: Supplementary file 1 [file molecules-24-00096-s001.pdf]

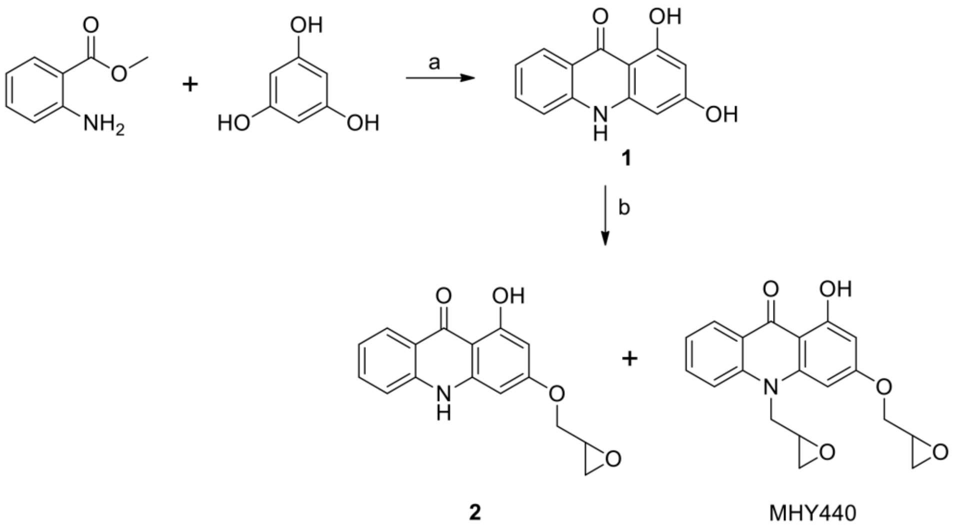

Scheme 1. Conditions and reagents: a) *p*-toluenesulfonic acid, cyclohexanol, reflux, 12 h, 58%, b) K_2CO_3, DMF, 65°C, 12 h, 16% for 2, and 20% for MHY440.
